# Tumor-associated macrophages in canine visceral hemangiosarcoma

**DOI:** 10.1177/03009858231179947

**Published:** 2023-06-21

**Authors:** Mikael Kerboeuf, Didrik Andreas Haugeberg, Tobias Olsen, Linn Kaia Sørling, Erling Olaf Koppang, Lars Moe, Anita Haug Haaland

**Affiliations:** 1 Norwegian University of Life Sciences, Ås, Norway

**Keywords:** cancer model, dog, immunohistochemistry, metastasis, M2, macrophage phenotype

## Abstract

Canine hemangiosarcoma (HSA) is a highly malignant tumor derived from hematopoietic stem cells and commonly occurs in visceral organs or skin. Visceral HSAs are particularly aggressive and progress rapidly despite multimodal treatment. Tumor-associated macrophages (TAMs) play a central role in carcinogenesis, tumor progression, and metastasis in humans and murine models. In this retrospective study, we investigated the prevalence and phenotype of TAMs in privately owned, treatment-naïve dogs with naturally occurring HSA. We used CD204 as a general macrophage marker and CD206 as a marker for M2-polarized macrophages. Formalin-fixed paraffin-embedded tissues from HSAs in the spleen (*n* = 9), heart (*n* = 6), and other locations (*n* = 12) from 17 dogs were sectioned and immunohistochemically labeled with CD204 and CD206 antibodies. The mean number of log(CD204)- and log(CD206)-positive cells and the ratio of log(CD206/CD204)-positive cells were compared with normal surrounding tissues and between tumor locations. There were significantly more macrophages and M2 macrophages, and a higher ratio of M2 macrophages to total macrophages in tumor hot spots (*P* = .0002, *P* < .0001, and *P* = .0002, respectively) and in tumor tissues outside of hot spots (*P* = .009, *P* = .002, and *P* = .007, respectively) than in normal surrounding tissues. There were no significant differences between tumor locations, but there was a trend toward higher numbers of CD204-positive macrophages within the splenic tumors. There was no association between histological parameters or clinical stage and TAM numbers or phenotype. As in humans, TAMs in dogs with HSA have a predominantly M2-skewed phenotype. Dogs with HSA could serve as excellent models to evaluate new TAM-reprogramming therapies.

Canine hemangiosarcoma (HSA) is a highly malignant tumor that is traditionally thought to derive from endothelial cells but is now believed to arise from hematopoietic stem cells.^17,29,52^ HSA can develop anywhere in the body but develops most often in the spleen, heart, liver, and skin.^8,16,18,49^ Visceral HSA is usually associated with early and widespread metastasis.^8,12,25,46,49^ Dogs often present with sudden onset of clinical signs caused by bleeding into body cavities, such as the peritoneal or pericardial cavities. Despite radical surgical treatment, dogs with visceral HSA quickly develop metastatic disease, with a median survival time (MST) of 4–8 weeks.^8,46,60^ However, in 2 population-based studies evaluating canine vascular tumors from the Norwegian Canine Cancer Register using a new histologic and immunohistochemical classification system based on the human classification scheme, overall survival was found to vary significantly between hemangioendotheliomas and HSAs, especially when they were located in the spleen.^12,15,16^ Hemangioendotheliomas were far less aggressive than poorly differentiated HSAs. In an attempt to develop a grading system, Ogilvie et al found that nuclear pleomorphism and mitotic scores were independently associated with disease-free interval (DFI) and survival time (ST) in dogs after complete tumor excision.^43^ Grade and other histological parameters were not significantly associated with DFI or ST. Additional intensive adjuvant chemotherapy prolongs STs in dogs with HSA, but the MST is still poor (4–6 months).^2,4,60^ Metronomic chemotherapy has produced similar effects on survival as traditional chemotherapy, but combining them does not further improve survival.^1,33^ In 1995, Vail et al showed that the immunostimulatory drug muramyl dipeptide (MDP) significantly improved the MST in dogs with splenic HSA when compared with dogs treated with surgery alone.^57^ Later MDP was found to activate the intracellular nucleotide-binding oligomerization domain-containing protein 2 (NOD2) receptor in macrophages, resulting in proinflammatory interleukin production.^42^ These interleukins reprogram macrophages toward an M1 polarization and result in antitumor activity.^13^

Tumor-associated macrophages (TAMs) play a central role in several aspects of cancer in humans and murine models.^23^ In vitro, macrophages undergo classical or alternative polarization into M1 and M2 macrophages in response to proinflammatory or antiinflammatory cues, respectively.^36^ M1 macrophages are associated with a proinflammatory Th1 response, while M2 are associated with an antiinflammatory Th2 response. M1 macrophages have a high production of proinflammatory interleukins, costimulatory molecules, and chemokines that can induce antigen-specific cytotoxic CD8 T-cell responses and chemotaxis to inflamed tissues.^44^ M2 macrophages, on the other hand, produce antiinflammatory interleukins, angiogenetic factors, and matrix remodeling enzymes vital for tissue repair.^10^ However, this M1/M2 dichotomy does not fully reflect the complexity of macrophage polarization and plasticity.^34,40^ In humans and mice, TAMs can exhibit both M1 and M2 associated traits, but are generally considered to be more M2-skewed due to their phenotypical and functional characteristics.^23,62^

TAMs have a central role in the tumor microenvironment (TME) and metastatic niche and are abundantly present in both.^23,62^ TAMs can promote tumor invasion, intravasation, tumor cell survival in circulation, and extravasation of tumor cells.^3,19,47^ They also promote tumor progression by inducing genetic instability, remodeling the extracellular matrix, promoting angiogenesis, and inhibiting the adaptive immune response.^23,62^ The number of TAMs in tumor tissue has an independent prognostic value in several human cancers, with higher numbers usually associated with worse outcomes. In dogs, however, few studies have described TAMs, M2 markers, and macrophage polarization.^5,14,21,22,45,50,59^ Reports indicate that higher numbers of infiltrating TAMs correlate with poor overall STs in dogs with mammary tumors.^50,51^ In metastatic lesions of dogs with HSA, the number of monocytes present within the tumor tissue was higher than in other tumors.^48^

Phenotypical macrophage markers are not necessarily transferable from one species to another and should be validated for each species.^41,55^ The mannose receptor CD206 is highly expressed on canine M2 macrophages in vitro, but little is known regarding its expression *in vivo*.^21^ Monteiro et al showed that almost all infiltrating macrophages in malignant canine mammary tumors were CD206-positive, further supporting the notion that TAMs are M2-skewed in dogs and have a central role in the tumor microenvironment.^38^ The scavenger receptor CD204 has been shown to be a good marker for canine macrophages, with no cross-reactivity with dendritic cells under normal conditions or undifferentiated blood monocytes.^27,56^

Due to the dismal prognosis despite multimodal treatment in canine visceral HSA, there is an urgent need for new treatment strategies. Immunotherapies aimed at repolarizing TAMs could represent such a new strategy. However, there are no published data on TAMs’ phenotypes in dogs with HSA. Our study aimed to investigate the number of CD204- (macrophages) and CD206 (M2-skewed macrophages)-positive cells in visceral HSA of privately owned, treatment naïve dogs. We wanted to study the prevalence of M2 macrophages within the tumor compared with surrounding tissue and between different tumor locations to assess if they are more abundantly present within the tumor tissues.

## Materials and Methods

### Study Population and Sample Collection

Tissue samples of all necropsy cases performed between January 2018 and December 2021 of privately owned, treatment-naïve dogs with naturally occurring visceral HSA, with and without disseminated disease, were collected retrospectively from the pathology archives of the Department of Preclinical Sciences and Pathology of the Norwegian University of Life Sciences. Corresponding clinical metadata (breed, age, sex, medication, and comorbidities) were obtained from the medical records. The owners’ written consent was required for the dogs to be included in the study. Dogs were excluded if they had undergone surgical treatment for HSA, had a history of occult atopic or allergic disease, had previous or concurrent neoplastic disease, were currently on immunomodulating medication (glucocorticoids or cytotoxic drugs), or had systemic inflammatory disorders (infectious or immune-mediated).

### Necropsy

There were 17 dogs included in the study (Table 1). All cases had undergone a standard necropsy performed by a veterinary pathologist. Detailed pathology records for each dog were available and included complete macroscopic and histologic descriptions of all major organ systems and any abnormal findings. Standard organ samples and samples from all tumors were collected and fixed in 10% buffered formalin, paraffin-embedded, and stored as formalin-fixed paraffin-embedded (FFPE) blocks at room temperature. There were 27 tumor samples in total, of which 13 were regarded as primary tumors and 14 as metastases in the pathology records. Seven cases (41.2%) had HSA located in 1 organ, while 10 (58.8%) had tumors in 2 or more locations. Since it was not always possible to establish which tumor represented the primary tumor, tumors were grouped into splenic, cardiac, and other locations.

**Table 1.** Overview of clinical characteristics, clinical stage, grade, location of tumor(s), and presence of tumors at several locations (yes/no) for dogs necropsied with visceral hemangiosarcoma based on necropsy findings and histopathology.

Case	Age (Years)	Breed	Sex	Stage	Tumor Present at Several Locations (Y/N)	Grade	Location of Tumors (Spleen/Heart/Other)
1	10	Finnish hound	Female	II	N	1	Spleen
2	10	Mix breed	Male	III	Y	1	Heart, lung
3	8	Golden retriever	Female	II	N	1	Spleen
4	8	Gordon setter	Male	III	Y	1	Spleen, heart
5	9	Golden retriever	Male	III	Y	1	Lung
6	10	Tibetan spaniel	Male	II	N	1	Spleen
7	9	Manchester terrier	Male	III	Y	2	Lung, omentum, peritoneum
8	9	Golden retriever	Female	III	Y	1	Spleen, heart, liver
9	11	Golden retriever	Male	III	Y	2	Lung, intramuscular (pelvis)
10	13	Gordon setter	Male	III	Y	1	Spleen, liver
11	6	Shorthaired vorster	Female	III	Y	2	Mesentery
12	8	Standard poodle	Female	III	Y	1	Kidney
13	8	Flat-coated retriever	Female	III	Y	2	Spleen, heart, liver
14	12	Kleiner münsterlander	Male	II	N	2	Spleen
15	11	Norfolk terrier	Male	II	N	1	Spleen
16	11	Beagle	Male	II	N	1	Heart
17	12	Mix breed	Female	II	N	1	Heart

### Staging and Histological Parameters

Hematoxylin and eosin-stained sections were reviewed by a board-certified veterinary pathologist to confirm the histopathological diagnosis and to assess the histological parameters of the tumors. Histological parameters were scored according to the grading system used by Ogilvie et al.^43^ Briefly, the overall differentiation was scored from 1 to 3, with 3 being the least differentiated, nuclear pleomorphism from 0 to 3, with 3 being marked nuclear pleomorphism, the amount of necrosis from 0 to 3 (assessed histologically after trimming of macroscopically visible necrotic areas before paraffin-embedding), with 3 being more than 50% necrosis, and the number of mitosis from 0 to 3, with 3 being above 30 mitoses per 10 high-power fields (HPFs) (400x, equivalent to a total area of 2.37 mm^2^). A total score was calculated, with 0–5 being graded as 1, 6–9 as Grade 2, and 10–12 as Grade 3. The necropsy reports were used retrospectively to assign a clinical stage for each case from I to III, according to the previously established staging scheme (Table 2).^58^

**Table 2.** Previously established three-tiered clinical staging scheme for dogs with hemangiosarcoma.^58^

Primary tumor (T)	
T0	No evidence of tumor
T1	Tumor less than 5 cm in diameter and confined to primary tissues
T2	Tumor 5 cm or greater or ruptured, invading subcutaneous tissues
T3	Tumor invading adjacent structures, including muscle
Regional lymph nodes (N)	
N0	No regional lymph node involvement
N1	Regional lymph node involvement
N2	Distal lymph node involvement
Distant metastasis (M)	
M0	No evidence of distant metastasis
M1	Distant metastasis
Stage	
I	T0 or T1, N0, M0
II	T1 or T2, N0 or N1, M0
III	T2 or T3, N0, N1 or N2, M1

**Table 3.** Overview of the nontransformed mean numbers of CD204- and CD206-positive cells per high-power field (area of 0.078 mm^2^) and the ratio of CD206/CD204-positive cells in the different counted regions, depending on tumor location, stage, and histological parameters (mitotic score, necrosis score, nuclear pleomorphism score, and differentiation score).

	Tumor Hot Spot	Tumor Tissues Outside of Hot Spots	Normal Surrounding Tissues
Location			
All locations	CD204: 68.6 CD206: 56.4 CD206/CD204: 0.91	CD204: 33.2 CD206: 19.6 CD206/CD204: 0.72	CD204: 34.5 CD206: 11.2 CD206/CD204: 0.51
Spleen	CD204: 83.8 CD206: 67.4 CD206/CD204: 0.98	CD204: 35.9 CD206: 17.3 CD206/CD204: 0.49	CD204: 56.2 CD206: 9.8 CD206/CD204: 0.16
Heart	CD204: 59.3 CD206: 49.7 CD206/CD204: 0.89	CD204: 34.0 CD206:19.8 CD206/CD204: 0.91	CD204: 18.1 CD206: 8.5 CD206/CD204: 0.42
Other	CD204: 56.0 CD206: 47.3 CD206/CD204: 0.82	CD204: 29.2 CD206: 22.6 CD206/CD204: 0.86	CD204: 25.2 CD206: 14.0 CD206/CD204: 0.87
Stage			
I	Na	Na	Na
II	CD204: 73.2 CD206: 53.5 CD206/CD204: 0.81	CD204: 29.8 CD206: 17.5 CD206/CD204: 0.81	CD204: 18.1 CD206: 0.8 CD206/CD204: 0.04
III	CD204: 67.0 CD206: 57.5 CD206/CD204: 0.94	CD204: 34.6 CD206: 20.5 CD206/CD204: 0.68	CD204: 36.7 CD206: 12.6 CD206/CD204: 0.58
Mitotic score			
0	CD204: 67.8 CD206: 58.4 CD206/CD204: 0.89	CD204: 32.1 CD206: 19.8 CD206/CD204: 0.74	CD204: 28.6 CD206: 11.5 CD206/CD204: 0.54
1	CD204: 74.7 CD206: 41.1 CD206/CD204: 1.01	CD204: 58 CD206: 15.0 CD206/CD204: 0.26	CD204: 128 CD206: 6.1 CD206/CD204: 0.05
2	Na	Na	Na
3	Na	Na	Na
Necrosis score			
0	CD204: 73.7 CD206: 56.2 CD206/CD204: 0.74	CD204: 35.0 CD206: 18.5 CD206/CD204: 0.58	CD204: 24.6 CD206: 9.0 CD206/CD204: 0.36
1	CD204: 70.1 CD206: 58.0 CD206/CD204: 1.00	CD204: 31.1 CD206: 18.9 CD206/CD204: 0.86	CD204: 52.3 CD206: 9.5 CD206/CD204: 0.11
2	CD204: 82.6 CD206: 53.0 CD206/CD204: 0.80	CD204: 38.5 CD206: 23.7 CD206/CD204: 0.65	CD204: 41.3 CD206: 14.7 CD206/CD204: 0.80
3	CD204: 51.6 CD206: 57.8 CD206/CD204: 1.08	CD204: 27.7 CD206: 18.4 CD206/CD204: 0.84	CD204: 11.5 CD206: 12.9 CD206/CD204: 1.06
Nuclear pleomorphism score			
0	CD204: 86.4 CD206: 62.3 CD206/CD204: 0.74	CD204: 36.6 CD206: 21.1 CD206/CD204:0.59	CD204: Na CD206: Na CD206/CD204: Na
1	CD204: 73.1 CD206: 41.3 CD206/CD204: 0.66	CD204: 32.0 CD206: 17.0 CD206/CD204: 0.69	CD204: 32.3 CD206: 13.1 CD206/CD204: 0.63
2	CD204: 62.1 CD206: 68.3 CD206/CD204: 1.14	CD204: 33.8 CD206: 22.1 CD206/CD204: 0.77	CD204: 36.9 CD206: 9.1 CD206/CD204: 0.38
3	Na	Na	Na
Differentiation score			
1	CD204: 70.0 CD206: 66.5 CD206/CD204: 0.96	CD204: 31.7 CD206: 21.7 CD206/CD204: 0.87	CD204: 36.2 CD206: 13.9 CD206/CD204: 0.71
2	CD204: 73.7 CD206: 37.3 CD206/CD204: 0.62	CD204: 36.3 CD206: 18.2 CD206/CD204: 0.54	CD204: 37.1 CD206: 11.2 CD206/CD204: 0.43
3	CD204: 53.9 CD206: 59.2 CD206/CD204: 1.29	CD204: 31.3 CD206: 11.2 CD206/CD204: 0.39	CD204: 18.1 CD206: 0.8 CD206/CD204: 0.04

Abbreviation: Na, not available.

## Immunohistochemistry

For the present study, new tissue sections were made from the FFPE blocks and immunohistochemically (IHC) labeled. Sequential sections were made from each block for labeling with different antibodies to reduce variability between sections. Tissues were sectioned at 4 µm thickness using a microtome and mounted on poly-lysin-coated SuperFrost^TM^ Plus slides (Thermo Fisher Scientific, Oslo, Norway). All washing steps were performed using 2 changes of phosphate-buffered saline (PBS), each for 5 minutes, and all incubations were performed in a moisture chamber on a rotating table at room temperature. Tissue sections were deparaffinized in xylene and rehydrated through an ethanol gradient using a standard protocol on an automated slide stainer. Heat-induced epitope retrieval was performed in a pressure cooker at 110^o^C for 10 minutes using Diva Decloaked (Biocare Medical, Histolab, Gothenburg, Sweden). Tissues were encircled using a water-repelling liquid blocker pen (PAP Pen, Dako, Glostrup, Denmark). Endogenous peroxidase activity was inhibited using Peroxidazed 1 (Biocare Medical, Histolab, Gothenburg, Sweden) for 10 minutes. The sections were blocked using Background Sniper (Biocare Medical, Histolab, Gothenburg, Sweden) for 10 minutes. The sections were then incubated with primary antibodies diluted in Da Vinci Diluent Green (Biocare Medical, Histolab, Gothenburg, Sweden) for 30 minutes. CD204 (mouse antihuman, clone SRA-E5, Abnova) was applied at a 1:100 dilution, and CD206 (rabbit antihuman, polyclonal, Abcam) at 1:800 dilution. Both antibodies have been validated in dogs for immunohistochemistry on FFPE tissues.^6,27,56^ Sections were incubated with Mach 1 mouse probe (Biocare Medical, Histolab, Gothenburg, Sweden) for CD204 for 15 minutes and Mach 1 universal probe (Biocare Medical, Histolab, Gothenburg, Sweden) for CD206 for 30 minutes. Sections were then incubated with 3-amino-9-ethylcarbazole (AEC) substrate chromogen (Biocare Medical, Histolab, Gothenburg, Sweden) for 10 minutes, then counterstained with Meyer’s hematoxylin for 10 seconds. Tissue sections were mounted using a water-soluble mounting medium (Aquatex^®^, Merck, Darmstadt, Germany) and left to dry overnight. Each tissue sample was stained with both CD204 and CD206 antibodies. Negative controls were processed by omitting primary antibodies. Isotype controls were labeled as part of the protocol optimization to ensure no unspecific labeling of the tissues. A sample from each tissue (spleen, heart, lung, peritoneum, liver, kidney, and muscle) was labeled using the same protocol while replacing the primary antibody with a species-specific isotype control at the same dilution (mouse IgG and rabbit IgG isotype controls, Invitrogen, Thermo Fisher Scientific, Oslo, Norway). Splenic tissue sections from a necropsied control dog without pathological findings were used as positive controls.

## Immunohistochemical Analysis and Quantification

All slides were manually scanned using a Zeiss AX10 microscope, equipped with a Zeiss axiocam 506 color camera, coupled with Zen pro 2012 (blue edition) image acquiring software (Carl Zeiss Microscopy GmbH, Jena, Germany). Sections that showed weak or no immunolabeling were excluded from the analysis. Areas with folded or detached tissues were also excluded. When present, areas of necrosis and hemorrhage were avoided. Within tumor tissues (*n* = 27), CD204- and CD206-positive cells were counted both in the areas with the highest densities of positive cells, known as hot spots, and outside the hot spots. Hot spots were identified subjectively by the investigators. Cells were counted in 5 randomly selected HPFs within the hot spots and similarly in the tumor tissues outside of the hot spots and in normal tissue surrounding the tumor and reported as a mean number per HPF. The area of a HPF was 0.078 mm^2^. If there was no normal tissue surrounding the tumor, positive cells were counted only within the tumor. The ratio of CD206-positive macrophages was estimated using the ratio of CD206-/CD204-positive cells.

## Statistical Analysis

All statistical analyses were performed using JMP pro 16.1.0 (SAS Institute Inc., Cary, NC). The number of CD204- and CD206-positive cells, as well as the ratio of CD206/CD204-positive cells were log-transformed, before being fitted into a mixed model, using the model building function in JMP. To compare these numbers between the different counted regions, tumor location and counted region were set as fixed factors, while case number and tissue sample number were set as random factors. When assessing differences between tumor locations, tumor location was set as a fixed factor, case number and tissue sample number were set as random factors and counted regions within the tumors were assessed individually. When assessing the effect of histological parameter scores on the counting data, the mitotic score, mitotic count, necrosis score, nuclear pleomorphism score, and differentiation score were set as fixed factors, whereas case number and tissue sample number were set as random factors and the counted regions within the tumors were assessed individually. When assessing the effect of clinical stage, stage was set as a fixed factor, case number and tissue sample number were set as random factors, and counted regions within the tumors were assessed individually. All models fulfilled the conditions for using mixed models (independence, homogeneity of variance, linearity, and normality of residuals) when assessed using graphical methods (residual by predicted plots and normal quantile plots of residuals). Tukey’s tests were used to compare differences between different fixed factors when there were more than 2 groups. *P*-values < .05 were considered statistically significant for the statistical testing.

## Results

### Clinical Characteristics and Tumor Samples

The mean age was 9.7 years (range: 6–13). There were 10 (58.8%) male and 7 (41.2%) female dogs. Seven (41.2%) dogs were classified as having Stage II and 10 (58.8%) dogs as having Stage III disease. Twelve (70.6%) of the tumors were graded as Grade 1 and 5 (29.4%) as Grade 2. None of the tumors were classified as grade 3. In 8 of the tumor samples, there was not enough surrounding normal tissue to quantify positive cells outside the tumor tissue. One tumor sample in each of 2 dogs (1 from a splenic tumor and 1 from a liver metastasis) had extensive necrosis, making it impossible to quantify immunolabeled cells. These tumors were excluded from the analysis, while the remaining tumors from these dogs were included.

### Quantification of CD204- and CD206-Positive Cells in Tumor Tissues and Normal Surrounding Tissues

**Figure 1.**
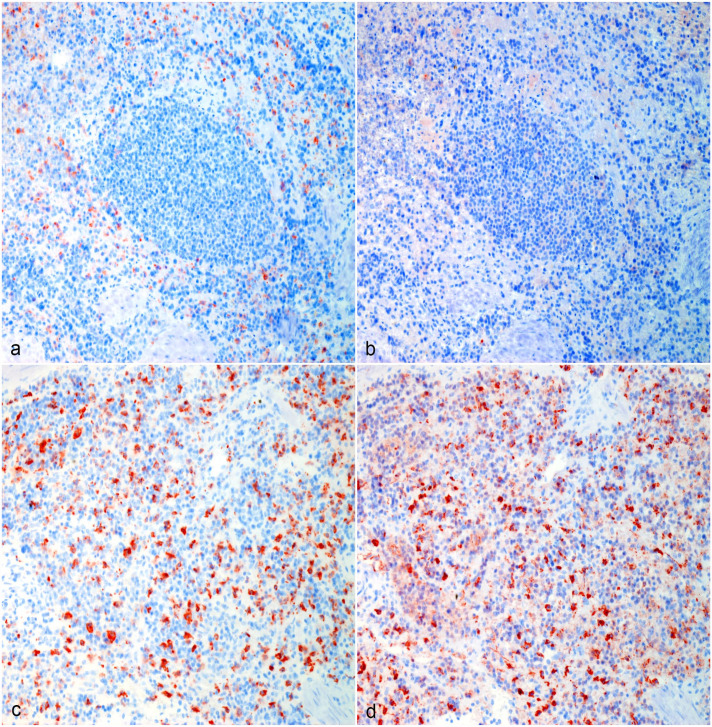
Hemangiosarcoma, spleen, dog. (a, b) Normal surrounding splenic tissue, Case 10. (a) CD204 immunolabeling of red pulp macrophages surrounding an area of white pulp. (b) The majority of red pulp macrophages are not immunolabeled for CD206. (c, d) Hemangiosarcoma macrophage hot spot, Case 10. (c) Strong CD204 immunolabeling of tumor-associated macrophages. (d) The majority of tumor-associated macrophages are immunolabeled for CD206. Formalin-fixed paraffin-embedded tissue, AEC chromogen and hematoxylin counterstain. Abbreviation: AEC, 3-amino-9-ethylcarbazole.

**Figure 2.**
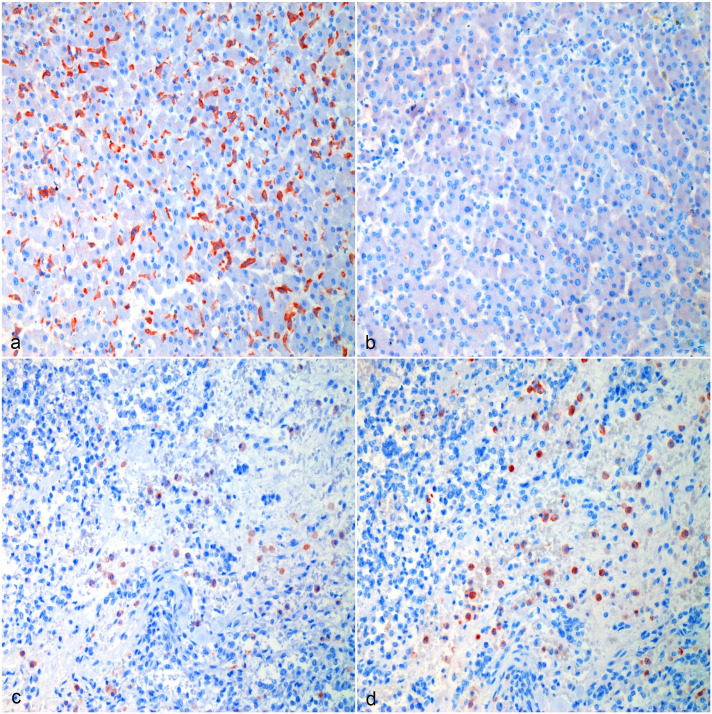
Hemangiosarcoma, liver, dog. (a, b) Normal surrounding hepatic tissue, Case 10. (a) CD204 immunolabeling of Kupffer cells. (b) Most tissue resident macrophages in the liver are not immunolabeled for CD206. (c, d) Hemangiosarcoma macrophage hot spot, Case 10. (c) Strong CD204 immunolabeling of tumor-associated macrophages in the liver. (d) The majority of tumor-associated macrophages in the liver are immunolabeled for CD206. Formalin-fixed paraffin-embedded tissue, AEC chromogen and hematoxylin counterstain. Abbreviation: AEC, 3-amino-9-ethylcarbazole.

**Figure 3.**
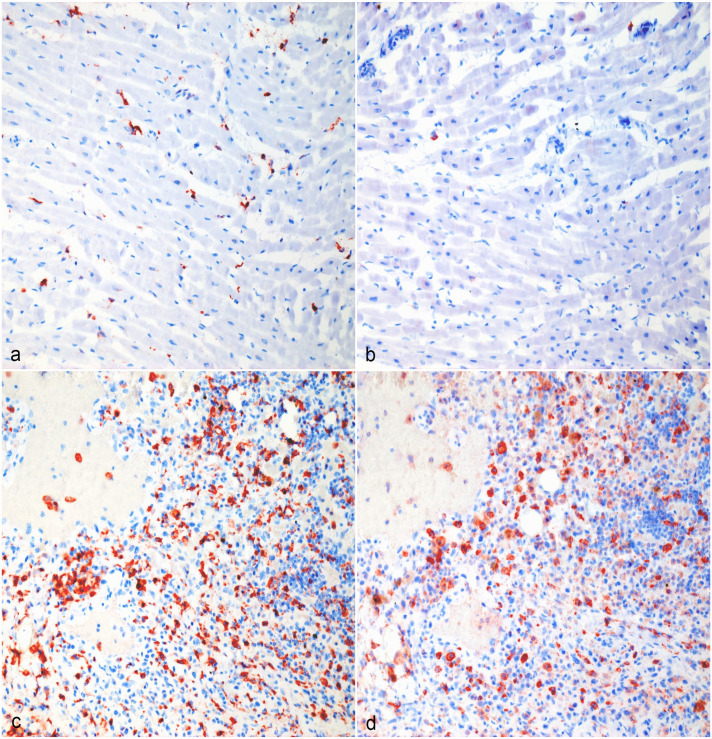
Hemangiosarcoma, heart, dog. (a, b) Normal surrounding cardiac tissue, Case 9. (a) CD204 immunolabeling of interstitial cardiac macrophages. (b) Many tissue-resident macrophages in the heart are not immunolabeled for CD206. (c, d) Hemangiosarcoma macrophage hot spot, Case 9. (c) Strong CD204 immunolabeling of tumor-associated macrophages in the heart. (d) The majority of tumor-associated macrophages in the heart are immunolabeled for CD206. Formalin-fixed paraffin-embedded tissue, AEC chromogen and hematoxylin counterstain. Abbreviation: AEC, 3-amino-9-ethylcarbazole.

**Figure 4.**
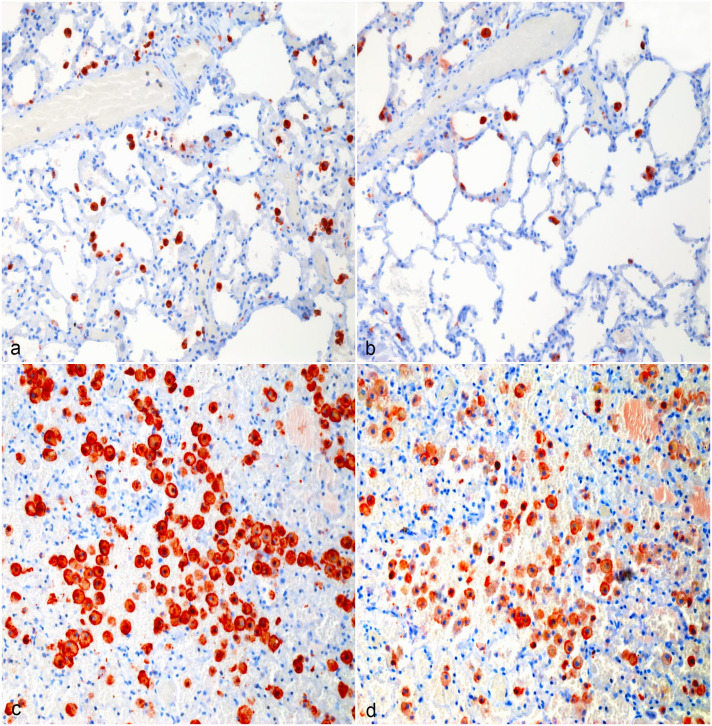
Hemangiosarcoma, lung, dog. (a, b) Normal surrounding pulmonary tissue, Case 4. (a) CD204 immunolabeling of interstitial and alveolar macrophages. (b) Most alveolar and interstitial macrophages in the lungs are also immunolabeled for CD206. (c, d) Hemangiosarcoma macrophage hot spot, Case 4. (c) Strong CD204 immunolabeling of tumor-associated macrophages in the lungs. (d) The majority of tumor-associated macrophages in the lungs are immunolabeled for CD206. Formalin-fixed paraffin-embedded tissue, AEC chromogen and hematoxylin counterstain. Abbreviation: AEC, 3-amino-9-ethylcarbazole.

**Figure 5.**
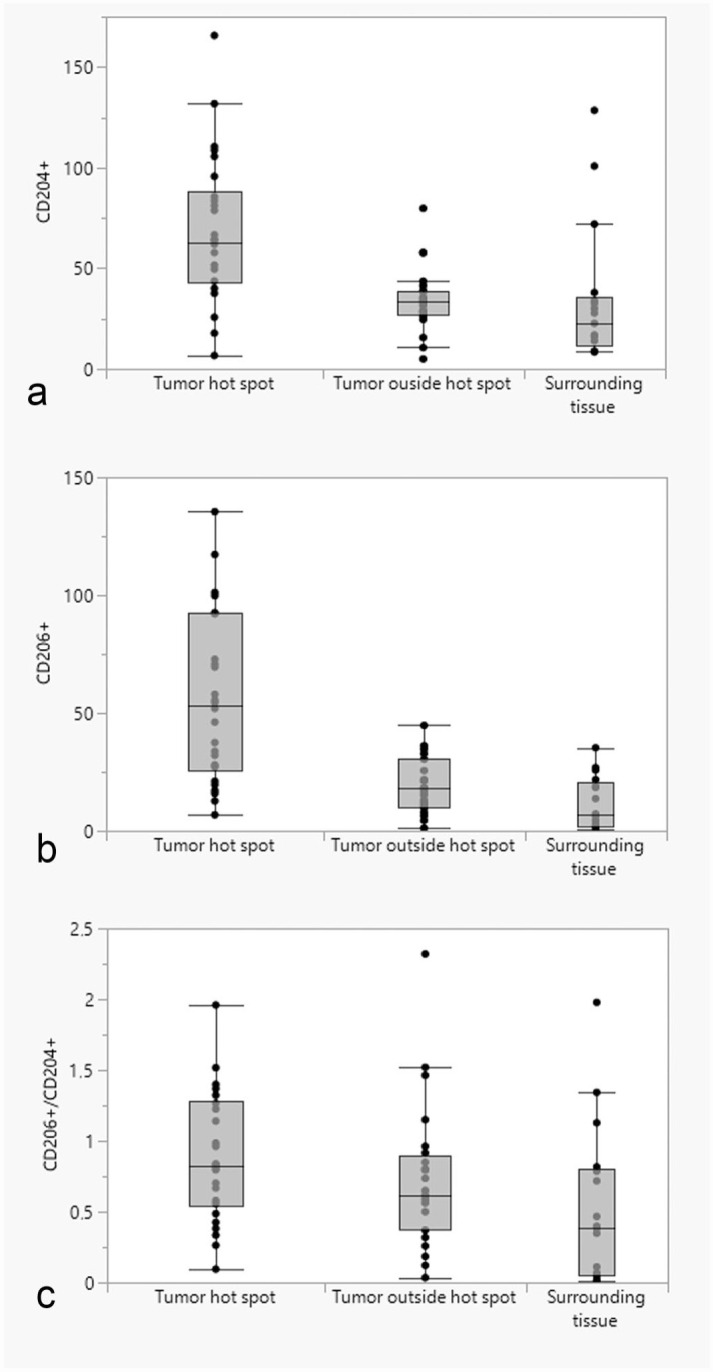
Box and whisker plots showing the differences in the median number of (a) CD204-positive cells; (b) CD206-positive cells; (c) ratio of CD206-/CD204-positive cells between the normal surrounding tissues, tumor-associated macrophage hot spots, and tumor tissues outside of hot spots.

Tissue-resident macrophages and tumor-infiltrating macrophages strongly immunolabeled for CD204 (Figs. 1a, 2a, 3a, 4a). There were areas with high densities of CD204-positive cells, forming hot spots within the tumors (Figs. 1c, 2c, 3c, 4c), but not in the surrounding tissue. Mean log(CD204) was significantly higher both within tumor hot spots and in the tumor outside of hot spots than in the normal surrounding tissues (*P* = .0002 and *P* = .009, respectively) (Fig. 5a). Mean log(CD204) was not significantly different between tumor hot spots and in the tumor outside of hot spots (*P* = .43).

Similarly, tumor-infiltrating macrophages strongly immunolabeled for CD206 (Figs. 1d, 2d, 3d, 4d). Tissue-resident macrophages in the surrounding normal tissue had more variable immunolabeling, depending on the organ (Figs. 1b, 2b, 3b, 4b). Interstitial and alveolar macrophages in the lungs were mostly CD206 positive, as were interstitial macrophages in the renal cortex. On the other hand, only a very small proportion of macrophages within the splenic red pulp were CD206 positive. Similarly, Kupffer cells in the liver were CD206-negative, with few interstitial macrophages or intravascular monocytes showing positive labeling in the liver. Interstitial macrophages in the heart were also mostly CD206-negative. Mean log(CD206) was significantly higher both within tumor hot spots and in the tumor outside of hot spots than in the normal surrounding tissues (*P* < .0001 and *P* = .002, respectively) (Fig. 5b). In addition, mean log(CD206) was significantly higher within tumor hot spot than in the tumor outside of hot spots (*P* = .001). The mean log(CD206/CD204) was also significantly higher both within tumor hot spots and in the tumor outside of hot spots than in the normal surrounding tissues (*P* = .0002 and *P* = .007, respectively) (Fig. 5c). Mean log(CD206/CD204) was not significantly different between tumor hot spots and in the tumor outside of hot spots (*P* = .45).

### Comparison of CD204- and CD206-Positive Cells Between Tumor Locations

**Figure 6.**
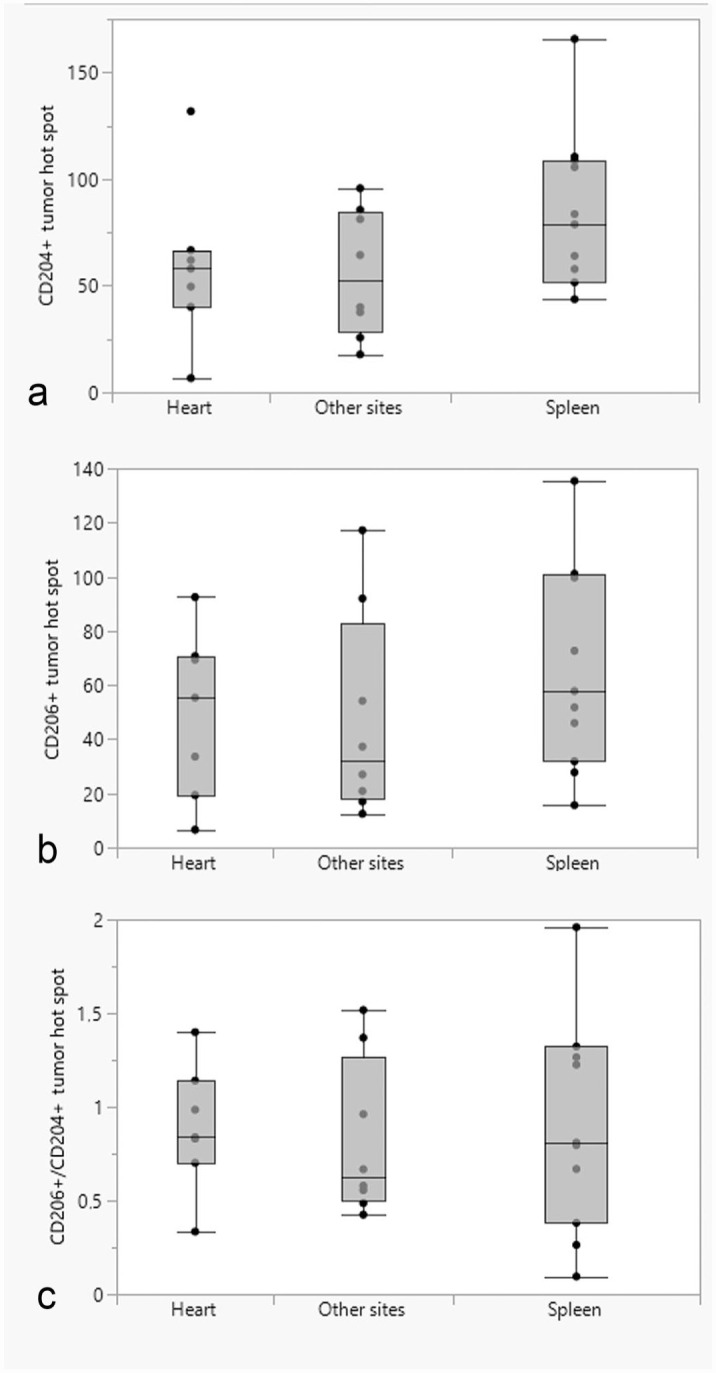
Box and whisker plots showing the differences in the median number of (a) CD204-positive cells; (b) CD206-positive cells; (c) ratio of CD206-/CD204-positive cells in tumor hot spots between different tumor locations (heart, spleen, and other sites).

**Figure 7.**
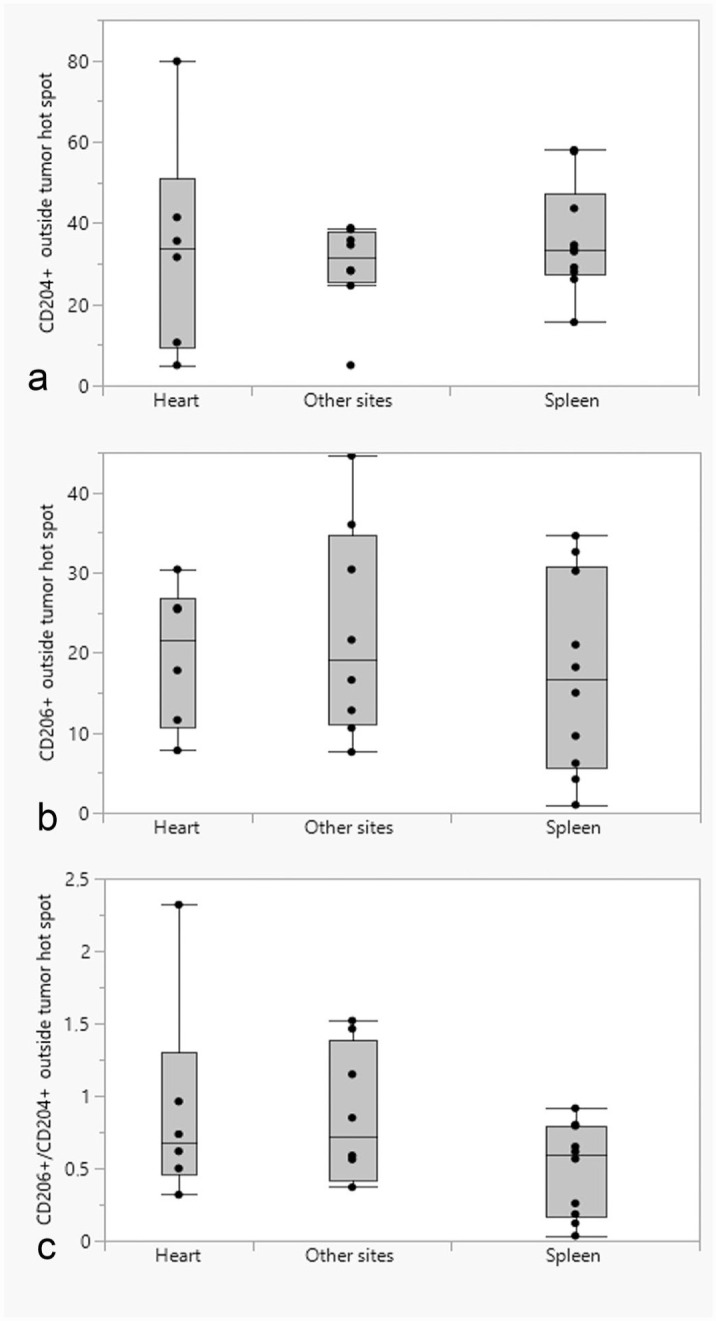
Box and whisker plots showing the differences in the median number of (a) CD204-positive cells; (b) CD206-positive cells; (c) ratio of CD206-/CD204-positive cells in tumor tissues outside of hot spots between different tumor locations (heart, spleen, and other sites).

The mean log(CD204) was not significantly different between tumor locations (spleen, heart, or other sites), either in tumor hot spots (*P* = .59) (Fig. 6a) or in tumor tissues outside hot spots (*P* = .70) (Fig. 7a). Similarly, the mean log(CD206) in hot spots (Fig. 6b) or in the tumor tissues outside of hot spots (Fig. 7b) were not significantly different between different tumor locations (*P* = .46 and *P* = .46, respectively). The mean log(CD206/CD204) was not significantly different between different tumor locations in either hot spots (*P* = .94) (Fig. 6c) or the tumor tissues outside of hot spots (*P* = .14) (Fig. 7c).

### Comparison of CD204- and CD206-Positive Cells Between Tumors of Different Stages and Histological Parameter Scores

The mean log(CD204) was significantly higher in tumor hot spots among tumors with a pleomorphism score of 0 than those with a score of 1 or 2 (*P* = .01 and *P* = .02, respectively). The mean log(CD204) in tumor tissues outside of hot spots was, however, not significantly different between tumors with different pleomorphism scores (*P* = .39). Mean log(CD204) in tumor hot spots and in the tumor tissues outside of hot spots did not vary significantly between different mitotic scores (*P* = .06 and *P* = .45), necrosis scores (*P* = .10 and *P* = .70), differentiation scores (*P* = .06 and *P* = .34), or show any correlation with mitotic count (*P* = .10 and *P* = .64). The mean log(CD206) in tumor hot spots and in the tumor tissues outside of hot spots did not vary significantly between mitotic scores (*P* = .85 and *P* = .25), necrosis score (*P* = .81 and *P* = .34), nuclear pleomorphism (*P* = .28 and *P* = .19), or differentiation scores (*P* = .45 and *P* = .70), or correlate with mitotic count (*P* = .37 and *P* = .19). Similarly, the mean log(CD206/CD204) in tumor hot spots and in the tumor tissues outside of hot spots did not vary significantly between mitotic scores (*P* = .27 and *P* = .65), necrosis score (*P* = .47 and *P* = .65), nuclear pleomorphism (*P* = .16 and *P* = .90), differentiation scores (*P* = .44 and *P* = .50), or correlate with mitotic count (*P* = .61 and *P* = .72).

Neither mean log(CD204), mean log(CD206), or mean log(CD206/CD204) in tumor hot spots were significantly different between tumors of different clinical stages (*P* = .40, *P* = .77, and *P* = .70, respectively). Similarly, mean log(CD204), mean log(CD206), or mean log(CD206/CD204) were not significantly different in tumor tissues outside of hot spots for different clinical stages (*P* = .26, *P* = .88, and *P* = .70, respectively).

## Discussion

We found that the mean ratio of CD206-/CD204-positive cells was significantly higher within tumor hot spots and in the tumor tissue outside of hot spots than in the surrounding normal tissues. Hence, there were more M2-skewed macrophages within the tumor tissue than outside, indicating an active protumoral polarization within the tumor. Our results are in line with those reported in human and murine tumors, suggesting TAM-targeting therapies may represent a viable treatment option for canine HSA.^23,62^

In this study, there were more CD204- and CD206-positive cells within tumor hot spots and in the tumor tissue outside of hot spots than in normal surrounding tissues (spleen, heart, liver, kidneys, and lungs). This finding supports the notion that there is either active recruitment of circulating monocytes that differentiate into macrophages and/or that local tissue-resident macrophages expand in response to the growth of HSA. In murine models of lung cancer, pancreatic cancer, and glioblastoma, TAMs originate from both expanding tissue-resident macrophages and circulating monocytes.^11,30,63^ Regan et al showed that canine HSA cells secreted high levels of the chemokine CCL2, stimulating monocyte migration and recruitment to metastases.^48^ However, they did not explore the relative contribution of the recruited monocytes to the total TAM number, nor can this be determined based on our results.

We found no significant differences in the numbers of CD204-positive or CD206-positive cells per HPF (0.078 mm^2^) between tumor locations, nor in the ratio of CD206-/CD204-positive cells. The number of CD204-positive cells per HPF was higher in the splenic tumors than in the tumors in the heart and other organs, but not significantly so. Knowing whether the splenic or cardiac lesion represents the primary tumor in dogs with HSA can be challenging, especially in a retrospective study design. Therefore, we divided the lesions into cardiac, splenic, and other sites. The fact that the number of CD204-positive cells per HPF was higher in the splenic lesions, which is the most commonly reported primary tumor site, could correspond to higher numbers of TAMs within the primary tumor compared with metastases.^4,8^ Given the small number of dogs, the study was probably underpowered to show any such difference. Similar results were reported in a study comparing the phenotypical differences between paired primary and metastatic tumor samples in humans with clear cell renal cell carcinoma.^39^ The number of CD204-positive cells seemed to be higher within primary tumors than in metastases. They also observed higher ratios of CD163 (an M2-associated marker in humans)-positive macrophages in the primary tumors compared with metastases. Interestingly, the number of ionized calcium-binding adaptor molecule 1 (IBA1)-positive cells, another macrophage marker, showed the opposite pattern. On the other hand, Withers et al showed that the number of CD204-positive macrophages in pulmonary metastases was significantly higher than in the primary tumors of dogs with osteosarcoma.^61^ Whether these differences in numbers of TAMs between primary and metastatic lesions are due to biological differences between tumor types, or investigated organs, remains unexplored.

Since our aim was to study macrophage phenotypes in treatment-naïve dogs, in the different tumor sites, we could not relate our findings to clinical outcomes. Most chemotherapies, including those used for treating dogs with HSA, can impact the number of TAMs and their phenotype.^9,20,32^ Chemotherapies will generally shift TAMs toward a proinflammatory, antitumoral M1 phenotype, which would probably have changed the results of our study. Furthermore, the acute stress associated with surgery can affect macrophage phenotypes and TAM infiltrates.^26,35,54^ Surgery has been shown to increase the numbers of TAMs within the primary tumor and metastases and skew TAMs and macrophages in the metastatic target organ toward a protumoral M2 phenotype.^26,35,54^

When comparing clinical stage and histological parameters with TAM numbers and phenotype, nuclear pleomorphism was the only score that showed a significant correlation. Higher pleomorphism scores were associated with lower numbers of CD204-positive cells. However, there were only 2 dogs with tumors having a pleomorphism score of 0, which puts the validity of this finding in question. Several studies have shown that higher TAMs numbers correlate with poor clinical outcomes in several human cancers.^62^ Similar findings are emerging within the veterinary field, with higher TAMs numbers correlating with either poor STs or high histological grades.^14,45,50,59^ Whether our findings reflect the fact that there is little association between TAM numbers and histological parameters in dogs with HSA or whether our study was underpowered remains unclear. Since most HSAs have similar biological behavior, with widespread metastasis and poor survival, there might not be any differences in TAM numbers and phenotype between tumors.^25,46,49^

Targeting TAMs in cancer therapy has gained a lot of interest in the last decade.^28,53^ Several strategies have been suggested and include either (1) depletion of TAMs, (2) inhibition of recruitment of blood monocytes, or (3) reprogramming of TAMs. Results from trials based on inhibition of recruitment and depletion of TAMs have been disappointing, so the focus seems to be changing toward reprogramming.^7^ The drug MDP was one of the earliest immunotherapies used in dogs.^31,57^ MDP had a significant clinical benefit in dogs with osteosarcoma and HSA compared with those treated with surgery alone. MDP binds to the intracellular NOD2 receptor of macrophages, dendritic cells, monocytes, and Paneth cells.^24,42^ NOD2 binding results in activation of the nuclear factor kappa B (NF-κB) pathway and downstream production of proinflammatory cytokines, such as tumor necrosis factor-α (TNF-α), interleukin (IL)-6, IL-8, and IL-12. These cytokines are usually associated with M1-skewed macrophages and improved tumoricidal abilities. The clinical benefit of MDP gives us hope that more selective TAM-targeting immunotherapies might improve the survival of dogs with HSA in the future.^57^

The expression of CD206 in normal tissue-resident macrophages in various organs has not been previously studied in dogs. Tissue-resident macrophages in the normal tissue surrounding the tumor in the liver, spleen, and heart were largely CD206-negative. Most alveolar and interstitial macrophages in the lungs were CD206-positive. Whether this is also the case in healthy dogs without cancer or was an effect of premetastatic niche formation remains unexplored. In humans, most alveolar macrophages of healthy individuals seem to be CD206 positive, which corresponds well with our findings.^37^ We also observed that tissue-resident macrophages in the kidney cortex were CD206 positive, but only 1 dog had renal metastasis.

Our study has several possible limitations. First, we did not include tissue samples from noncancer patients as a control group, although we included the healthy surrounding tissues from the same dogs as an internal control. The number and phenotype of macrophages in the healthy surrounding tissues may have been influenced by the tumor and could be different under physiological conditions in healthy dogs. Second, the study was retrospective, and the representativity of the included cases could have been improved. There is also a risk that relevant clinical and histopathological data were missed during data collection. Third, the number of included dogs was small, and there is a risk that differences in macrophage numbers and phenotype in HSA lesions between different locations were missed. Finally, performing CD204 and CD206 immunohistochemistry on separate slides instead of using methods like multiplex IHC/immunofluorescence introduces some errors when estimating double-positive cells. Although staining sequential sections should result in limited variation, counting the exact same areas is impossible.

In conclusion, we have shown that the number of CD206-expressing M2-polarized macrophages was significantly higher within tumor tissues than in the normal surrounding tissues. This is in line with findings in human medicine and supports the notion that TAMs are immunosuppressive and protumoral. We found no significant differences in the number or phenotype of macrophages related to the location of tumors. There was no association between clinical staging or histologic parameters and the numbers or phenotype of TAMs, except for higher nuclear pleomorphism scores being associated with lower TAM numbers. Given the translational value of dogs with cancer, dogs with HSA could serve as a good model to evaluate new therapies aimed at reprogramming TAMs.
